# Variants in the 14q32 miRNA cluster are associated with osteosarcoma risk in the Spanish population

**DOI:** 10.1038/s41598-018-33712-4

**Published:** 2018-10-18

**Authors:** Idoia Martin-Guerrero, Nerea Bilbao-Aldaiturriaga, Angela Gutierrez-Camino, Borja Santos-Zorrozua, Vita Dolžan, Ana Patiño-Garcia, Africa Garcia-Orad

**Affiliations:** 1 Department of Genetics, Physical Anthropology and Animal Physiology, Faculty of Science and Technology, University of the Basque Country, UPV/EHU, Leioa, Spain; 2 Department of Genetics, Physical Anthropology and Animal Physiology, Faculty of Medicine and Nursery, UPV/EHU, Leioa, Spain; 3 Institute of Biochemistry, Faculty of Medicine, Ljubljana, Slovenia; 4 Laboratory of Pediatrics, University Clinic of Navarra, Pamplona, Spain; 5 BioCruces Health Research Institute, Barakaldo, Spain

## Abstract

Association studies in osteosarcoma risk found significant results in intergenic regions, suggesting that regions which do not codify for proteins could play an important role. The deregulation of microRNAs (miRNAs) has been already associated with osteosarcoma. Consequently, genetic variants affecting miRNA function could be associated with risk. This study aimed to evaluate the involvement of all genetic variants in pre-miRNAs described so far in relationship to the risk of osteosarcoma. We analyzed a total of 213 genetic variants in 206 pre-miRNAs in two cohorts of osteosarcoma patients (n = 100) and their corresponding controls (n = 256) from Spanish and Slovenian populations, using Goldengate Veracode technology (Illumina). Four polymorphisms in pre-miRNAs at 14q32 miRNA cluster were associated with osteosarcoma risk in the Spanish population (rs12894467, rs61992671, rs58834075 and rs12879262). Pathway enrichment analysis including target genes of these miRNAs pointed out the WNT signaling pathways overrepresented. Moreover, different single nucleotide polymorphism (SNP) effects between the two populations included were observed, suggesting the existence of population differences. In conclusion, 14q32 miRNA cluster seems to be a hotspot for osteosarcoma susceptibility in the Spanish population, but not in the Slovenian, which supports the idea of the existence of population differences in developing this disease.

## Introduction

Osteosarcoma is the most common primary malignant bone tumor, mainly occurring in children and adolescents. The precise etiology of the disease remains partially unknown^1^, but genetic factors seem to play a key role in its pathogenesis^2,3^. To date, several case-control studies have reported associations of common genetic variants with osteosarcoma risk^3–5^, but these studies were mainly focused on regions codifying for proteins, because results are easily interpreted biologically. However, a genome wide association study (GWAS) in osteosarcoma showed that 8 out of the 13 most significant genetic variants were located in regions with no clear functional consequence^6^, results that are more difficult to interpret. Similar results were found in other GWAS in different cancer types, in which 44% of significant signals were described to be located in intergenic regions^7^. All these data together suggest that regions which do not codify for proteins could play an important role in the risk of cancer, in general, and in osteosarcoma, in particular. One of the most studied non-coding RNAs are microRNAs (miRNAs), molecules of 20 nucleotides that regulate gene expression at the post-transcriptional level by binding to the 3′ untranslated region (UTR) of a target mRNA^8^, leading to its translation inhibition or degradation. Through this mechanism, miRNAs can regulate more than half of human genes^9^. More than 600 miRNAs have been proposed to be involved in osteogenesis regulation^10^, so it is reasonable to think that miRNAs deregulation can be linked to osteosarcoma susceptibility. In fact, alterations of miR-34c affecting Notch signaling pathway were associated with the pathogenesis of osteosarcoma^11^, and the deregulation of the 14q32 miRNA cluster was also linked to the progression and prognosis of osteosarcoma^12^. Genetic variations in miRNAs can alter their function affecting their gene targets. These variants can modify the miRNA expression levels if they are located in the pre-miRNA or the mRNA-miRNA binding if they are located in the *seed* region. Consequently, genetic variations in pre-miRNAs affecting their function could be involved in the risk of cancer. Several works have already described polymorphisms in miRNAs associated with the susceptibility to different types of cancer^13,14^. Despite all these evidences, few studies have analyzed the involvement of miRNA single nucleotide polymorphism (SNPs) in the risk of osteosarcoma so far. Although only a low number of SNPs were analyzed, significant results were found with two variations belonging to miR-34 family^15,16^ and with one located in mir-124a^17^.

Considering that the number of annotated miRNAs has increased substantially up to 2500 miRNAs approximately^18^, the aim of this study was to evaluate the contribution in the risk of osteosarcoma of variants in pre-miRNAs. With that objective, all variants in pre-miRNAs with a minor allele frequency (MAF) higher than 1% were analyzed in a representative group of osteosarcoma patients from two populations.

## Materials and Methods

### Patients

The study population included 100 patients (<34 years) diagnosed of osteosarcoma at the Oncology Unit of the Department of Pediatrics of the University Clinic of Navarra (n = 74) between 1985 and 2003 and University Children’s Hospital of Liubliana (n = 26) between 1990 and 2008. Both patient cohorts were residents in Spain or Slovenia at the moment of diagnosis and had West European ancestry. Moreover, 256 healthy individuals of European origin with no previous history of cancer (n = 160 and n = 96 from Spain and Slovenia, respectively) were added (Table 1). Informed consent was obtained from all patients or their parents before sample collection. The study was approved by the Spanish Ethics Committees for Clinical Research of Euskadi (CEIC-E) (CEISH/102R/2011/GARCIA-ORAD CARLES 67/02/12) and the University of Navarra (105/2009), and by the Slovenian Ethics Committee for Research in Medicine (bilateral project BI- ES/04-05-016) and was carried out according to the Declaration of Helsinki.

**Table 1:** Study population.

	Total	Controls	Cases
**Participants (n)**	356	256	100
**Population (n;%)**			
Spain	234	160 (68.37)	74 (31.62)
Slovenia	122	96 (78.68)	26 (21.31)
**Age (mean; sd)**			
Spain		69.01 (17.5)	14.5 (4.7)
Slovenia		46 (9.3)	19.5 (8.6)
**Sex (f/m)** ^**a**^			
Spain	111/120	81/79	30/41
Slovenia	51/71	35/58	13/13

Abbreviations: n, number of individuals; sd, standard deviation; f, female; m, male.

^a^Sex was not available for all patients.

### Selection of polymorphisms in miRNAs

We selected all the pre-miRNAs including SNPs with a MAF > 0.01 in European/Caucasian populations described in the databases until May 2014. Since, on the one hand, osteosarcoma is a polygenic disease in which associated genes are not totally defined, and, on the other hand, a single miRNA can regulate several transcripts which are not completely known nowadays, we decided to analyze all polymorphic miRNAs to date. MAF > 0.01 was selected because this frequency was required to detect significant differences in our sample size.

The SNP selection was performed using miRNA SNiPer (www.integratomics-time.com/miRNA-SNiPer/), NCBI and literature review. Finally, a total of 213 SNPs in 206 pre-miRNAs were included.

### Genotyping

Peripheral blood samples were obtained as the source of DNA from Spanish patients and all healthy controls, while in Slovenian osteosarcoma patients DNA was extracted from the areas of formalin fixed paraffin embedded (FFPE) material verified by an experienced pathologist to be representative of normal tissue. Most FFPE samples were osteogenic (>96%) from histological point of view, and all of them were primary malignancy. Genomic DNA was extracted using standard procedures^19^. DNA was quantified using PicoGreen (Invitrogen Corp., Carlsbad, CA). For each sample, 400 ng of DNA were genotyped using the GoldenGate Genotyping Assay with Veracode technology according to the published Illumina protocol. Data were analyzed with Genome Studio software for genotype clustering and calling. As quality control, duplicate samples and CEPH trios (Coriell Cell Repository, Camden, NJ) were genotyped across the plates, following the Illumina recommendations.

### Statistical analysis

The association between genetic polymorphisms and the risk of osteosarcoma was evaluated by the χ2 or Fisher’s exact test. The effect sizes of the associations were estimated by the OR’s from univariate logistic regression. The most significant test among codominant, dominant, recessive and additive was used to determine the statistical significance of each SNP. The results were adjusted for multiple comparisons by the False Discovery Rate (FDR)^20^. In all cases the significance level was set at 5%. Analyses were performed by using R v2.11 software. χ2 test was used to search for any deviation of Hardy-Weinberg equilibrium (HWE) in controls. Those SNPs that showed deviations from HWE in control population were removed from the analyses.

### Bioinformatic analyses

#### miRNAs secondary structures prediction

The RNAfold web tool (http://rna.tbi.univie.ac.at) was used to calculate the minimum free energy secondary structures and to predict the most stable secondary structures of the miRNAs showing significant SNPs.

#### miRNAs expression analyses

Expression levels of miRNAs were analyzed in a series of 18 osteosarcoma cell lines (<34 years) and 4 normal bone samples, using data publicly available in GEO database under the accession GSE28423^21^. T-tests were performed using the GEO2R web tool, applying a Benjamini and Hochberg FDR adjusted p-value cut-off of 0.05.

#### Gene targets selection and pathways analysis

MiRWalk^22^ (http://zmf.umm.uni-heidelberg.de/apps/zmf/miRwalk2/) database was used to select miRNA targets. Only targets predicted by at least 8 different algorithms provided by miRWalk were selected. Enriched pathway analyses of putative target genes were determined with ConsensusPath database (CPdB) (http://consensuspathdb.org/)^23^ using the over-representation analysis module. Gene list were analyzed against the default collection of KEGG^24^, Reactome^25^ and BioCarta (http://cgap.nci.nih.gov/Pathways/BioCarta_Pathways) pathway databases. A conservative *p*-value cutoff (0.001) was used.

## Results

### Genotyping results

Genotyping analyses were performed in 100 patients diagnosed of osteosarcoma (74 Spanish and 26 Slovenian) and 256 cancer-free controls (160 and 96, respectively). Successful genotyping was obtained in 350 of 356 DNA samples (98.3%). Finally, a total of 140 SNPs were included in the association analyses, after eliminating SNPs with genotyping failures (<80%), monomorphic in the studied populations, or with deviations from HWE in controls (Table S1).

### Genotype association study

We found 23 SNPs significantly associated with osteosarcoma risk; 14 SNPs in 14 miRNAs in the Spanish population and 9 SNPs in 8 miRNAs in the Slovenian. When the two populations were analyzed together, 11 SNPs at 11 miRNAs were significant.

**Figure 1:**
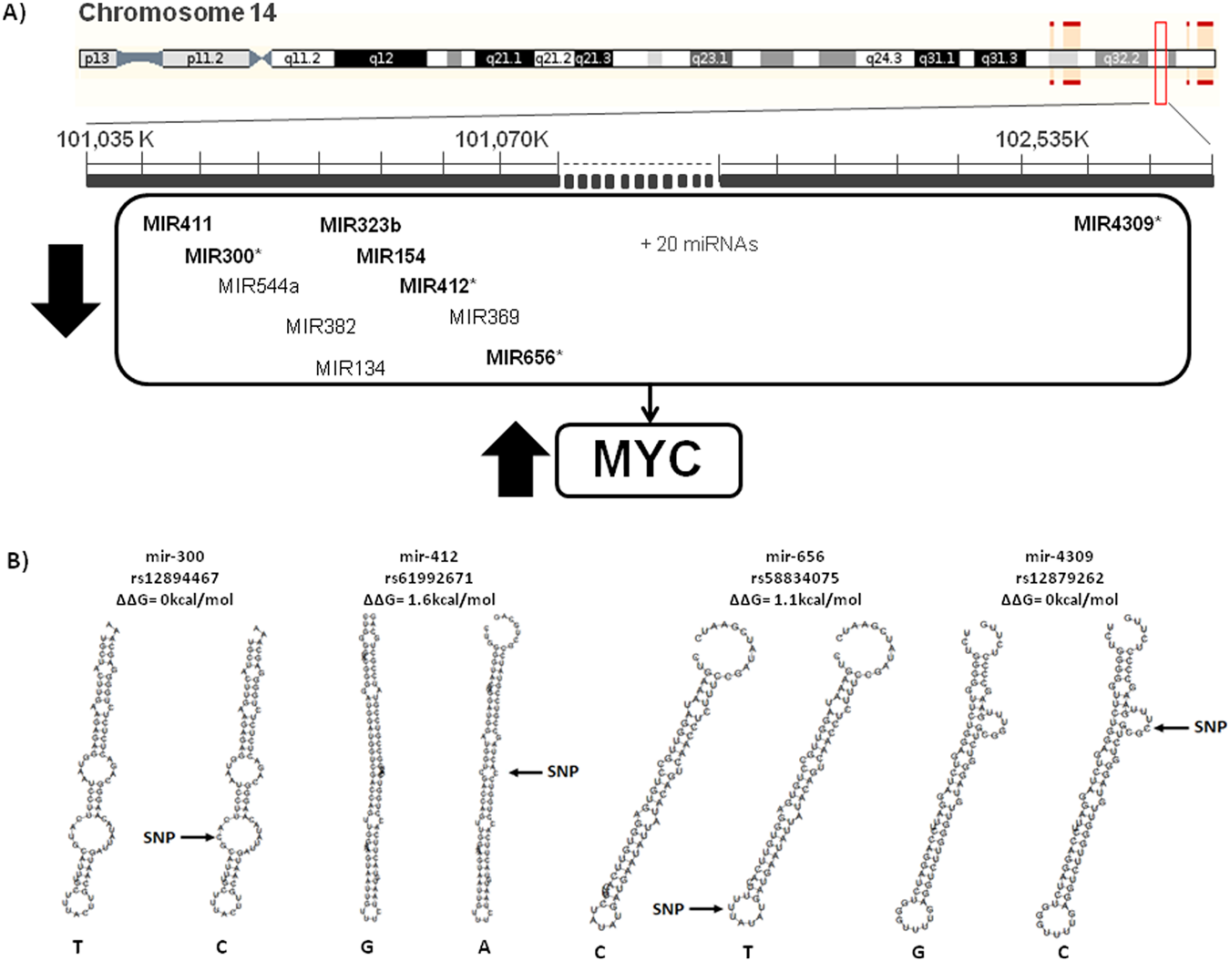
14q32 miRNA cluster. (**A**) Diagram of the 14q32 miRNA cluster, including miRNAs analyzed in our study (in bold), miRNAs with significant SNPs (highlighted with an asterisk), and miRNAs described in the literature to be downregulated. (**B**) Secondary structures of the 14q32 miRNAs showing significant SNPs, predicted by RNAfold web tool.

In the Spanish population, 4 out of 14 significant SNPs were located at 14q32 region (Fig. 1). Among them, rs12894467 at miR-300 showed the most significant association value under the log-additive model (CC vs CT vs TT). The frequency of TT genotype was found to be 2.5 times higher in patients than in controls (OR = 2.01, 95% CI: 1.32–3.06; P = 0.001). With regard to the other three significant SNPs at 14q32 region, we found an increase in the risk of osteosarcoma for the genotypes AG + AA for rs61992671, CT for rs58834075 and CG for rs12879262 located at miR-412, miR-656 and miR-4309, respectively (OR = 2.21, OR = 4.98 and OR = 1.99). Other 10 SNPs showed statistically significant results (P < 0.05), 6 located in pre-miRNAs, 2 in mature miRNAs and 1 in the seed region (Table 2). After FDR correction, no SNP remained significant.

**Table 2:** Polymorphisms in miRNAs associated with osteosarcoma risk in the Spanish population.

	SNP	miRNA	Localization	Genotype	N (%)controls N = 160	N (%)cases N = 69	OR (CI 95%)	P
1	rs12894467	mir-300	14q32 pre-miRNA	CC	74 (46.2)	19 (27.5)	2.01 (1.32–3.06)	0.001 (add)
				CT	71 (44.4)	34 (49.3)		
				TT	15 (9.4)	16 (23.2)		
2	rs356125	mir-2278	9q22 pre-miRNA	GG	140 (87.5)	68 (98.6)	1	0.002 (codom)
				AG	20 (12.5)	1 (1.4)	0.1 (0.01–0.78)	
				AA	0 (0	0 (0)	0.00 (0.00)	
3	rs77639117	mir-576	4q25 pre-miRNA	AA	156 (97.5)	60 (87.0)	1	0.003 (codom)
				AT	4 (2.5)	9 (13.0)	5.85 (1.74–19.71)	
				TT	0 (0)	0 (0)	0.00 (0.00)	
4	rs7247237	mir-3188	19p13 pre-miRNA	CC	72 (45.3)	23 (34.3)	1	0.004 (rec)
				CT	77 (48.4)	31 (46.3)	3.59 (1.49–8.66)	
				TT	10 (6.3)	13 (19.4)		
5	rs60871950	mir-4467	7q22.1 miRNA	GG	35 (22.0)	27 (39.1)	1	0.009 (dom)
				AG	83 (52.2)	23 (33.3)	0.44 (0.24–0.81)	
				AA	41 (25.8)	19 (27.5)		
6	rs10505168	mir-2053	8q23.3 pre-miRNA	AA	78 (49.1)	25 (36.2)	1	0.009 (codom)
				AG	65 (40.9)	42 (60.9)	2.02 (1.11–3.65)	
				GG	16 (10.1)	2 (2.9)	0.39 (0.08–1.81)	
7	rs61992671	mir-412	14q32 miRNA	GG	57 (35.6)	13 (20.0)	1	0.018 (dom)
				AG	66 (41.2)	35 (53.8)	2.21 (1.11–4.41)	
				AA	37 (23.1)	17 (26.2)		
8	rs58834075	mir-656	14q32 pre-miRNA	CC	157 (98.1)	63 (91.3)	1	0.021 (codom)
				CT	3 (1.9)	6 (8.7)	4.98 (1.21–20.55)	
				TT	0 (0)	0 (0)		
9	rs10406069	mir-5196	19q13 pre-miRNA	GG	110 (69.6)	43 (62.3)	1	0.021 (codom)
				AG	39 (24.7)	26 (37.7)	1.71 (0.93–3.13)	
				AA	9 (5.7)	0 (0.0)	0.00 (0.00)	
10	rs12879262	mir-4309	14q32 pre-miRNA	GG	111 (69.8)	39 (56.5)	1	0.022 (codom)
				CG	43 (27.0)	30 (43.5)	1.99 (1.10–3.59)	
				CC	5 (3.1)	0 (0.0)	0.00 (0.00)	
11	rs702742	mir-378h	5q33 pre-miRNA	AA	117 (73.1)	58 (86.6)	1	0.022 (dom)
				AG	41 (25.6)	8 (11.9)	0.42 (0.19–0.93)	
				GG	2 (1.2)	1 (1.5)		
12	rs10422347	mir-4745	19p13 miRNA	CC	138 (87.3)	51 (75.0)	1	0.025 (dom)
				CT	19 (12.0)	17 (25.0)	2.30 (1.12–4.73)	
				TT	1 (0.6)	0 (0.0)		
13	rs2289030	mir-492	12q22 pre-miRNA	CC	130 (81.2)	60 (87.0)	1	0.026 (rec)
				CG	30 (18.8)	6 (8.7)	0.00	
				GG	0 (0.0)	3 (4.3)		
14	rs35770269	mir-449c	5q11 seed	AA	61 (38.4)	35 (50.7)	0.64 (0.42–0.98)	0.038 (add)
				AT	71 (44.7)	28 (40.6)		
				TT	27 (17.0)	6 (8.7)		

Abbreviations: OR, Odd Ratio; CI, Confidence Interval; add, additive; codom, codominant; dom, dominant; rec, recessive.

In the Slovenian population, 9 SNPs were significant. Among them, rs35613341 at miR-5189 showed the most significant association. The genotype CG for rs35613341 showed a protective effect (OR = 0.07, 95% CI: 0.01–0.59; under codominant model), association that remained significant after FDR correction. Another genotype in the same miRNA (AG + AA for rs56292801) also showed protective effect (OR = 0.25; 95% CI:0.08–0.80). Other 7 SNPs displayed significant results (P < 0.05), 4 located in pre-miRNAs and 3 in the seed region (Table 3).

**Table 3:** Polymorphisms in miRNAs associated with osteosarcoma risk in the Slovenian population.

N	SNP	miRNA	Localization	Genotype	N (%) controls N = 96	N(%) cases N = 25	OR (CI 95%)	P
1	rs35613341	mir-5189	16q24 pre-miRNA	CC	49 (51.0)	16 (69.6)	1	0.0001* (codom)
				CG	41 (42.7)	1 (4.3)	0.07 (0.01–0.59)	
				GG	6 (6.2)	6 (26.1)	3.06 (0.86–10.85)	
2	rs4674470	mir-4268	2q35 pre-miRNA	TT	50 (52.1)	17 (85.0)	1	0.002 (codom)
				CT	40 (41.7)	1 (5.0)	0.07 (0.01 0.58)	
				CC	6 (6.2)	2 (10.0)	0.98 (0.18 5.33)	
3	rs2070960	mir-3620	1q42 seed	CC	75 (78.9)	21 (87.5)	1	0.008 (codom)
				CT	20 (21.1)	1 (4.2)	0.18 (0.02–1.41)	
				TT	0 (0.0)	2 (8.3)	0	
4	rs56292801	mir-5189	16q24 pre-miRNA	GG	51 (53.1)	18 (81.8)	1	0.010 (dom)
				AG	41 (42.7)	3 (13.6)	0.25 (0.08–0.80)	
				AA	4 (4.2)	1 (4.5)		
5	rs2273626	mir-4707	14q11 seed	AA	31 (32.3)	2 (8.7)	1	0.013 (dom)
				AC	45 (46.9)	15 (65.2)	5.01 (1.10–22.72)	
				CC	20 (20.8)	6 (26.1)		
6	rs6726779	mir-4431	2p16 pre-miRNA	TT	34 (35.8)	12 (63.2)	1	0.027 (codom)
				CT	51 (53.7)	4 (21.1)	0.22 (0.07–0.75)	
				CC	10 (10.5)	3 (15.8)	0.85 (0.20–3.62)	
7	rs9877402	mir-5680	8q22 pre-miRNA	AA	88 (93.6)	17 (85.0)	1	0.030 (rec)
				AG	6 (6.4)	1 (5.0)	0	
				GG	0 (0.0)	2 (10.0)		
8	rs243080	mir-4432	2p16 pre-miRNA	CC	30 (31.6)	12 (57.1)	1	0.030 (dom)
				CT	49 (51.6)	5 (23.8)	0.35 (0.13–0.91)	
				TT	16 (16.8)	4 (19.0)		
9	rs3746444	mir-499a	20q11 seed	TT	64 (66.7)	18 (75.0)	1	0.044 (rec)
				CT	30 (31.2)	3 (12.5)	6.71 (1.06–42.73)	
				CC	2 (2.1)	3 (12.5)		

*Significant after FDR correction. Abbreviations: OR, Odd Ratio; CI, Confidence Interval; add, additive; codom, codominant; dom, dominant; rec, recessive.

None of the miRNAs significant in the Spanish population were significant in the Slovenian.

In the global analysis, a total of 11 significant SNPs were detected. Nine of them had been already found significant in the Spanish or in the Slovenian populations. Among them, 3 SNPs showed more significant and 5 less significant P values in the total population than those found in each population separately. The other 3 out of 11 significant associations detected were new (Table S2). From the total of significant SNPs observed in the Spanish (n = 14) or in the Slovenian population (n = 9), 14 did not show significant results when both population were analyzed together.

### miRNAs secondary structures

We analyzed *in silico* the energy change (|ΔΔG|) and the secondary structures of the miRNAs with significant SNPs. In the Spanish population, 4/14 miRNAs showed drastic energy changes (>2.0 Kcal/mol) and 7 showed altered secondary structure (Fig. S1). With regard to the SNPs at 14q32 region, rs61992671 in miR-412 and rs58834075 in miR-656 induced positive energy changes which turned the miRNA hairpins from a stable to an unstable status. In the Slovenian population, 2/9 miRNAs showed energy changes >2.0 Kcal/mol and 3 displayed secondary structure changes (Fig. S2). In the global analysis, 2 of the 3 new detected miRNAs showed energy changes >2.0 Kcal/mol and all of them showed changes in the secondary structure (Fig. S3).

### miRNA expression

We studied the expression levels of miRNAs of interest in osteosarcoma cell lines using the public database Gene Expression Omnibus (GEO). Out of 22 miRNAs with significant SNPs, 5 miRNAs were represented in the GSE28423 database (miR-300, miR-412, miR-492, miR-576 and miR-656). From them, mir-300 was found significantly down-regulated in osteosarcoma cell lines group (logFC = −1.545; adj-p = 0.006). The rest of miRNAs showed no significant results (p > 0.05).

### Pathway analysis

We performed a pathway enrichment analysis with miRNAs of 14q32 region that modified the secondary structure, miR-412 and miR-656, using miRWalk database and ConsensusPathDB web tool. MiR-300 (the most significant SNP) was also included in pathway enrichment analysis although no remarkable results were observed (data not shown). For miR-412, we found two pathways over-represented, being both WNT signaling predicted by KEGG and Biocarta (Table S3). Regarding miR-656, only Ca2+ pathway was over-represented, with 7/55 genes targeted by this miRNA (Table S4). Of these 7 genes, 5 overlapped with WNT signaling pathway. When both miRNAs were analyzed together, 5 pathways were over-represented, being WNT signaling pathway the most significant (p = 0.000177) (Table S5), with 16/143 genes targeted by miR-412 and miR-656 (Table S6).

## Discussion

In the Spanish population, the most interesting result was that 4 genetic variants in miRNAs belonging to the 14q32 miRNA cluster were statistically associated with the risk of osteosarcoma. From these, rs12894467 T allele at miR-300 showed the most significant result, conferring a 2.01-fold increased risk. This polymorphism was also found significant when Spanish and Slovenian populations were analyzed together, what means that it showed the same trend in both cohorts (although it was not significant in the Slovenian sample individually). The other 3 significant SNPs of the cluster in the Spanish population (rs61992671, rs58834075 and rs12879262 at miR-412, miR-656 and miR-4309, respectively) were also associated with an increased risk of osteosarcoma. Interestingly, miRNAs of this cluster were found to be under-expressed in osteosarcoma in previous studies^26,27^. This miRNAs downregulation was correlated with *MYC* overexpression, that it is known to be related to the development of osteosarcoma^27^. The miRNAs down-expression was confirmed for mir-300 in a series of osteosarcoma cell lines using GEO dataset GSE28423. Moreover, the block of 14q32 miRNAs was shown to increase the tumorigenic potential in osteoblasts, suggesting that they could work as tumor suppressors. Consequently, the loss of function of these miRNAs could be considered as a causative factor in osteosarcomagenesis^27^. Supporting this idea, the bioinformatical analysis predicted that the SNPs in miR-412 and miR-656 decreased the stability of the miRNA hairpins, which has been suggested that may reduce the product of the mature miRNA^28^. This reduction in miRNA levels could increase the expression of their target genes. Interestingly, pathway analyses pointed out the WNT pathway as the most over-represented pathway, which is known to play an important role in osteoblastogenesis^29^. Other authors have also pointed out the involvement of WNT pathway in the development of osteosarcoma^30,31^. Dysregulation of Wnt signaling pathway allows β-catenin to accumulate and translocate into the nucleus, where it activates downstream oncogenes including *MYC*^32^. Considering these previous studies, we can hypothesize that variations in the pre-miRNAs miR-300, miR-412, miR-656 or miR-4309 could lead to their downregulation, altering the Wnt pathway which ultimately would lead to the overexpression of *MYC*. All these results would support the hypothesis that this region is a hotspot for the development of osteosarcoma. In fact, recent studies in early-onset osteosarcoma have shown that inherited imprinting defects in14q32 region affects gene and miRNA expression in this area, which could be associated with the pathobiology of osteosarcoma^33^.

Another interesting result in the Spanish population was found for rs35770269, located in the seed region of miR-449c. In this case, the T allele was observed to decrease the risk of osteosarcoma (OR = 0.64). This allele was proposed to alter the secondary structure of the miRNA (*in silico*), so the T allele could have a double action in the miRNA, one affecting its levels and another, the miRNA-mRNA binding. Of note, miR-449c is part of the highly conserved miR-449 cluster belonging to the miR-34 family^34^, a key regulator of tumor suppression^35^. SNPs in the miR-34 family had already been found involved in the risk of osteosarcoma: rs4938723 C and rs72631823 A were associated with a reduction of miR-34b and miR-34a, respectively^15,16^. In addition, the underexpression of miR-34a was shown to downregulate the suppression of the proto-oncogene C-*MET*, promoting osteosarcoma cell proliferation and migration^16^. Since miRNAs belonging to the same family usually share target genes, we can hypothesize that rs35770269 could affect the binding of miR-449c to *MET*.

The other 9 significant miRNA variants detected in the Spanish population also showed a putative effect on target genes with known involvement in osteosarcoma. For instance, rs77639117 T allele could increase the risk of osteosarcoma through upregulating miR-576, which in turn might downregulate *RB1*, a tumor suppressor gene inactivated in 35% of osteosarcoma patients^1^. The genotype rs2289030 GG could alter miR-492, affecting its target *PTEN*. This gene was previously shown to be downregulated in osteosarcoma cells^36–38^. Rs6087195 could alter the expression levels of miR-4467, which consequently could alter the expression of its putative target gene *SF1*, involved in DNA reparation function^39^. In this case, the miRNA dysfunction could be explained by a modification of the pre-miRNA secondary structure and a drastic energy change (3.9 Kcal/mol), which has been suggested to affect the stability of the miRNA^28^.

In the Slovenian population, rs35613341 and rs56292801 (both located at miR-5189) showed the most remarkable results. In this case, the significant association was caused by a decrease of the percentage of heterozygotes and an increase of the percentage of homozygotes. This fact suggests the presence of a deletion in this region in which a copy number variation (CNV) (according to the database of Genomic Variations) has been described. To the best of our knowledge, this is the first time that this CNV is associated with osteosarcoma risk. Another interesting finding was observed for rs3746444 located in the seed region of the pre-miR-499. The GG genotype was associated with increased risk of osteosarcoma. Similar results were observed in two previous meta-analyses studying the involvement of this polymorphism in cancer susceptibility in Caucasians (although not significant)^40,41^.

When both populations were analyzed together, a total of 6 SNPs increased the significance of association with respect to the individual analyses. These results indicate that all these SNPs showed the same trend in both populations, so they could be considered as disease markers. Among them, rs2910164 at miR-146a was previously associated with diverse types of cancer^42,43^. This SNP was also analyzed in relation to the risk of osteosarcoma in Chinese, showing the same trend as in our population (but it was not significant)^16^. When a meta-analysis including the three populations (Chinese, Spanish and Slovenian) was performed, a significant association was found under the dominant model (P = 0.003). The CG + CC rs2910164 genotype showed an OR = 0.57 (95% CI: 0.39–0.83) (Fig. S4). However, 5 SNPs decreased their significance level, what means that opposite results were detected in the two populations. This suggests that these SNPs are population specific, which indicates remarkable population differences in factors contributing to osteosarcoma risk.

This study has some limitations that might be addressed, such as the limited sample size. Nevertheless, considering the scarcity of the disease, we think that the number of patients included in the present study was enough to obtain valid results. Another possible weakness of the study was the relatively high failure rate in genotyping technique. However, this high chance of failure was accepted from the beginning, because despite the predicted problem with the technique, no other design option to amplify these polymorphisms was possible.

In conclusion, the most important findings of the present study indicated that SNPs located at the 14q32 miRNA cluster can be involved in the susceptibility of osteosarcoma in the Spanish population, confirming the interest of this region in the disease. Our results also confirm the existence of population differences in the risk of developing osteosarcoma. To our knowledge, this is the first study analyzing in depth so many SNPs at miRNAs in relation with the risk of osteosarcoma, which opens a promising approach to search for new susceptibility markers in this disease. New large-scale studies including functional analyses will help to validate our findings.

### Ethics approval and consent to participate

All procedures performed in studies involving human participants were in accordance with the ethical standards of the institutional and/or national research committee and with the 1964 Helsinki declaration and its later amendments or comparable ethical standards.

## Electronic supplementary material

Supplementary information
