# Supplementary material for: Variants in the 14q32 miRNA cluster are associated with osteosarcoma risk in the Spanish population
Source: Sci Rep. 2018 Oct 18;8:15414. doi: 10.1038/s41598-018-33712-4 (PMC6194014; doi:10.1038/s41598-018-33712-4)

**Variants in the 14q32 miRNA cluster are associated with osteosarcoma risk in the Spanish population**

Idoia Martin-Guerrero^1*^, Nerea Bilbao-Aldaiturriaga^2*^, Angela Gutierrez-Camino^2^ Borja Santos-Zorrozua^2^, Vita Dolžan^3^, Ana Patiño-Garcia^4^, and Africa Garcia-Orad^2,5^

| **Supplementary Table S1**. SNPs selected for the study after eliminating SNPs with genotyping failures, monomorphic in the studied populations, or with deviations from HWE in controls. | | | | | | | | |
| --- | --- | --- | --- | --- | --- | --- | --- | --- |
| **N** | **SNP** | **Gene** | **Location** | **% Genotyping** | **MAF (Population source)** | **MAF** | **Alleles** | **Exclusion criteria** |
| 1 | **rs1414273** | hsa-mir-548ac | 1p13.1 | 0.0 | 0.133 (Pilot CEU) | - | T:T | Genotyping failure |
| 2 | **rs74904371** | hsa-mir-2682 | 1p21.3 | 100.0 | 0.025 (Pilot CEU) | 0.03 | C:T |  |
| 3 | **rs12402181** | hsa-mir-3117 | 1p31.3 | 99.4 | 0.125 (Pilot CEU) | 0.131 | G:A |  |
| 4 | **rs521188** | hsa-mir-3671 | 1p31.3 | 100.0 | 0.025 (Pilot CEU) | 0.038 | A:G |  |
| 5 | **rs17111728** | hsa-mir-4422 | 1p32.3 | 99.7 | 0.06 (Pilot CEU) | 0.071 | T:C |  |
| 6 | **rs45530340** | hsa-mir-6084 | 1p36.12 | 99.7 | 0.175 (Pilot CEU) | 0.0 | C:C | Monomorphic |
| 7 | **rs72646786** | hsa-mir-3972 | 1p36.13 | 99.4 | 0.142 (Pilot CEU) | 0.118 | C:T |  |
| 8 | **rs56088671** | hsa-mir-4424 | 1q25.2 | 0.0 | 0.058 (Pilot CEU | - | T:T | Genotyping failure |
| 9 | **rs7522956** | hsa-mir-4742 | 1q42.11 | 99.2 | 0.248 | 0.233 | A:C |  |
| 10 | **rs2070960** | hsa-mir-3620 | 1q42.13 | 98.0 | 0.042 (Pilot CEU) | 0.081 | C:T |  |
| 11 | **rs701213** | hsa-mir-4427 | 1q42.2 | 0.0 | 0.237 (Pilot CEU) | - | T:T | Genotyping failure |
| 12 | **rs877722** | hsa-mir-4671 | 1q42.2 | 99.7 | 0.127 | 0.125 | A:T |  |
| 13 | **rs12473206** | hsa-mir-4433 | 2p14 | 0.0 | 0.267 (Pilot CEU) | - | T:T | Genotyping failure |
| 14 | **rs243080** | hsa-mir-4432 | 2p16.1 | 98.3 | 0.458 | 0.435 | C:T |  |
| 15 | **rs41291179** | hsa-mir-216a | 2p16.1 | 99.4 | 0.033 | 0.044 | A:T |  |
| 16 | **rs6726779** | hsa-mir-4431 | 2p16.2 | 98.0 | 0.35 (Pilot CEU) | 0.364 | T:C |  |
| 17 | **rs58450758** | hsa-mir-559 | 2p21 | 0.0 | 0.083 (Pilot CEU) | - | T:T | Genotyping failure |
| 18 | **rs62154973** | hsa-mir-4772 | 2q12.1 | 99.2 | 0.108 (Pilot CEU) | 0.106 | C:T |  |
| 19 | **seq_rs163642** | MIR4436B2 | 2q13 | 0.0 | 0.266 | - | T:T | Genotyping failure |
| 20 | **rs6430498** | hsa-mir-3679 | 2q21.2 | 98.9 | 0.3 (Pilot CEU) | 0.341 | G:A |  |
| 21 | **rs10173558** | mir-1302-4 | 2q33.3 | 100.0 | 0.208 (Pilot CEU) | 0.119 | T:C |  |
| 22 | **rs2241347** | hsa-mir-3130-1 | 2q33.3 | 0.0 | 0.125 (Pilot CEU) | - | T:T | Genotyping failure |
| 23 | **rs4674470** | hsa-mir-4268 | 2q35 | 98.3 | 0.158 | 0.21 | T:C |  |
| 24 | **rs74949342** | hsa-mir-5702 | 2q36.3 | 99.7 | 0.042 (Pilot CEU) | 0.003 | C:G |  |
| 25 | **rs78832554** | hsa-mir-4786 | 2q37.2 | 99.7 | 0.083 | 0.027 | G:A |  |
| 26 | **rs2292832** | hsa-mir-149 | 2q37.3 | 0.0 | 0.27 | - | T:T | Genotyping failure |
| 27 | **rs2292181** | hsa-mir-564 | 3p21.31 | 99.4 | 0.100 (Pilot CEU) | 0.053 | G:C |  |
| 28 | **rs11714172** | hsa-mir-4792 | 3p24.2 | 98.9 | 0.375 (Pilot CEU) | 0.351 | T:G |  |
| 29 | **rs6787734** | hsa-mir-3135a | 3p24.3 | 0.0 | 0.15 (Pilot CEU) | - | T:T | Genotyping failure |
| 30 | **rs9877402** | hsa-mir-5680 | 3q13.33 | 82.7 | 0.017 | 0.045 | A:G |  |
| 31 | **rs10934682** | hsa-mir-544b | 3q21.2 | 99.2 | 0.150 | 0.156 | T:G |  |
| 32 | **rs11713052** | hsa-mir-5092 | 3q21.2 | 99.4 | 0.025 | 0.028 | C:G |  |
| 33 | **rs78790512** | hsa-mir-6083 | 3q21.2 | 99.7 | 0.233 (Pilot CEU) | 0.162 | G:A |  |
| 34 | **rs9842591** | hsa-mir-5186 | 3q25.1 | 85.0 | 0.434 | 0.462 | C:A |  |
| 35 | **rs78831152** | hsa-mir-4789 | 3q26.31 | 98.9 | 0.092 (Pilot CEU) | 0.093 | C:T |  |
| 36 | **rs75715827** | hsa-mir-944 | 3q28 | 98.9 | 0.075 (Pilot CEU) | 0.074 | T:C |  |
| 37 | **rs6841938** | hsa-mir-1255b-1 | 4p14 | 0.0 | 0.058 (Pilot CEU) | - | T:T | Genotyping failure |
| 38 | **rs12512664** | hsa-mir-4274 | 4p16.1 | 97.5 | 0.342 (Pilot CEU) | 0.451 | A:G |  |
| 39 | **rs28645567** | hsa-mir-378d-1 | 4p16.2 | 99.2 | 0.05 (Pilot CEU) | 0.02 | G:A |  |
| 40 | **rs1077020** | hsa-mir-943 | 4p16.3 | 0.0 | 0.212 | - | T:T | Genotyping failure |
| 41 | **rs73239138** | hsa-mir-1269a | 4q13.2 | 98.9 | 0.258 | 0.236 | G:A |  |
| 42 | **rs28664200** | hsa-mir-1255a | 4q24 | 81.3 | 0.358 (Pilot CEU) | 0.265 | T:C |  |
| 43 | **rs77639117** | hsa-mir-576 | 4q25 | 100.0 | 0.025 (Pilot CEU) | 0.027 | A:T |  |
| 44 | **rs34115976** | hsa-mir-577 | 4q26 | 99.7 | 0.200 (Pilot CEU) | 0.195 | C:G | No HWE Spain |
| 45 | **rs257095** | hsa-mir-4636 | 5p15.31 | 99.7 | 0.119 | 0.134 | A:G | No HWE Spain |
| 46 | **rs12523324** | hsa-mir-4277 | 5p15.33 | 0.0 | 0.317 | - | T:T | Genotyping failure |
| 47 | **rs78541299** | hsa-mir-6075 | 5p15.33 | 100.0 | 0.025 (Pilot CEU) | 0.006 | G:A |  |
| 48 | **rs10061133** | hsa-mir-449b | 5q11.2 | 99.4 | 0.075 (Pilot CEU) | 0.088 | A:G |  |
| 49 | **rs10461441** | hsa-mir-548ae-2 | 5q11.2 | 0.0 | 0.067 (Pilot CEU) | - | T:T | Genotyping failure |
| 50 | **rs35770269** | hsa-mir-449c | 5q11.2 | 98.6 | 0.292 | 0.356 | A:T |  |
| 51 | **rs73112689** | hsa-mir-4459 | 5q11.2 | 0.0 | 0.15 (Pilot CEU) | - | T:T | Genotyping failure |
| 52 | **rs266435** | hsa-mir-4804 | 5q13.2 | 98.9 | 0.183 (Pilot CEU) | 0.119 | C:G |  |
| 53 | **rs79512808** | hsa-mir-3976 | 5q14.2 | 100.0 | 0.05 (Pilot CEU) | 0.011 | T:G |  |
| 54 | **rs367805** | hsa-mir-3936 | 5q31.1 | 98.0 | 0.267 | 0.319 | G:A |  |
| 55 | **rs2042253** | hsa-mir-5197 | 5q31.3 | 99.4 | 0.225 | 0.226 | A:G |  |
| 56 | **rs77055126** | hsa-mir-1303 | 5q32.2 | 0.0 | 0.025 (Pilot CEU) | - | T:T | Genotyping failure |
| 57 | **rs3734050** | hsa-mir-6499 | 5q33.1 | 98.9 | 0.111 | 0.046 | C:T |  |
| 58 | **rs13186787** | hsa-mir-1294 | 5q33.2 | 0.0 | 0.017 | - | T:T | Genotyping failure |
| 59 | **rs702742** | hsa-mir-378h | 5q33.2 | 99.2 | 0.111 | 0.101 | A:G |  |
| 60 | **rs936581** | hsa-mir-3141 | 5q33.2 | 99.4 | 0.221 | 0.204 | G:A |  |
| 61 | **rs62376935** | hsa-mir-585 | 5q35.1 | 99.4 | 0.042 (Pilot CEU) | 0.048 | C:T | No HWE Slovenia |
| 62 | **rs7709117** | hsa-mir-4634 | 5q35.2 | 98.9 | 0.467 (Pilot CEU) | 0.443 | A:G |  |
| 63 | **rs67182313** | hsa-mir-4642 | 6p21.1 | 98.9 | 0.208 (Pilot CEU) | 0.162 | A:G |  |
| 64 | **rs4285314** | hsa-mir-3135b | 6p21.33 | 0.0 | 0.425 (Pilot CEU) | - | T:T | Genotyping failure |
| 65 | **rs17737028** | hsa-mir-3143 | 6p22.1 | 100.0 | 0.013 | 0.008 | A:G |  |
| 66 | **rs12197631** | hsa-mir-548a-1 | 6p22.3 | 0.0 | 0.050 (Pilot CEU) | - | T:T | Genotyping failure |
| 67 | **rs9295535** | hsa-mir-5689 | 6p24.3 | 0.0 | 0.190 | - | T:T | Genotyping failure |
| 68 | **rs68035463** | hsa-mir-3144 | 6q22.31 | 98.9 | 0.283 (Pilot CEU) | 0.193 | C:A |  |
| 69 | **rs6977967** | hsa-mir-3683 | 7p22.1 | 99.4 | 0.142 (Pilot CEU) | 0.214 | A:G |  |
| 70 | **rs72631831** | hsa-mir-323b | 7p22.3 | 99.7 | 0.011 (miRNA panel) | 0.0 | G:G | Monomorphic |
| 71 | **rs850108** | hsa-mir-550a-3 | 7q14.3 | 0.0 | 0.2 (Pilot CEU) | - | T:T | Genotyping failure |
| 72 | **rs11983381** | hsa-mir-4653 | 7q22.1 | 98.3 | 0.158 (Pilot CEU) | 0.154 | A:G |  |
| 73 | **rs3823658** | hsa-mir-5090 | 7q22.1 | 99.4 | 0.1 (Pilot CEU) | 0.142 | G:A |  |
| 74 | **rs60871950** | hsa-mir-4467 | 7q22.1 | 99.2 | 0.417 (Pilot CEU) | 0.454 | G:A |  |
| 75 | **rs72631827** | hsa-mir-106b | 7q22.1 | 100.0 | 0.012 (miRNA panel) | 0.0 | G:G | Monomorphic |
| 76 | **rs41274239** | hsa-mir-96 | 7q32.2 | 100.0 | 0.017 (Pilot CEU) | 0.001 | A:G |  |
| 77 | **rs72631833** | hsa-mir-183 | 7q32.2 | 100.0 | 0.016 (miRNA panel) | 0.0 | G:G | Monomorphic |
| 78 | **rs76481776** | hsa-mir-182 | 7q32.2 | 99.4 | 0.067 (Pilot CEU) | 0.084 | C:T |  |
| 79 | **rs4909237** | hsa-mir-595 | 7q36.3 | 99.7 | 0.133 (Pilot CEU) | 0.169 | C:T |  |
| 80 | **rs80128580** | hsa-mir-5707 | 7q36.3 | 99.4 | 0.025 (Pilot CEU) | 0.03 | G:A |  |
| 81 | **rs66683138** | hsa-mir-3622a | 8p21.1 | 0.0 | 0.217 (Pilot CEU) | - | T:T | Genotyping failure |
| 82 | **rs73235381** | hsa-mir-548h-4 | 8p21.2 | 0.0 | 0.025 (Pilot CEU) | - | T:T | Genotyping failure |
| 83 | **rs79397096** | hsa-mir-597 | 8p23.1 | 100.0 | 0.033 (Pilot CEU) | 0.011 | G:A |  |
| 84 | **rs61388742** | hsa-mir-596 | 8p23.3 | 99.4 | 0.083 (Pilot CEU) | 0.098 | T:C |  |
| 85 | **rs487571** | hsa-mir-5680 | 8q22.3 | 0.0 | 0.383 (Pilot CEU) | - | T:T | Genotyping failure |
| 86 | **rs10505168** | hsa-mir-2053 | 8q23.3 | 98.6 | 0.292 | 0.31 | A:G |  |
| 87 | **rs2114358** | hsa-mir-1206 | 8q24.21 | 98.6 | 0.417 | 0.422 | T:C |  |
| 88 | **rs2648841** | hsa-mir-1208 | 8q24.21 | 99.2 | 0.058 (Pilot CEU) | 0.111 | C:A |  |
| 89 | **rs6997249** | hsa-mir-3686 | 8q24.21 | 0.0 | 0.258 | - | T:T | Genotyping failure |
| 90 | **rs28655823** | hsa-mir-4472-1 | 8q24.3 | 90.7 | 0.083 | 0.138 | G:C |  |
| 91 | **rs74428911** | hsa-mir-4474 | 9p21.3 | 100.0 | 0.017 (Pilot CEU) | 0.007 | G:T |  |
| 92 | **rs13299349** | hsa-mir-3152 | 9p22.1 | 84.1 | 0.275 | 0.37 | G:A | No HWE Spain |
| 93 | **rs75019967** | hsa-mir-4477a | 9q13 | 99.7 | 0.5 | 0.0 | A:A | Monomorphic |
| 94 | **rs67339585** | MIR3910-1, MIR3910-2 | 9q22.31 | 0.0 | 0.067 | - | T:T | Genotyping failure |
| 95 | **rs356125** | hsa-mir-2278 | 9q22.32 | 100.0 | 0.067 | 0.052 | G:A |  |
| 96 | **rs56195815** | hsa-mir-548aw | 9q34.13 | 0.0 | 0.200 (Pilot CEU) | - | T:T | Genotyping failure |
| 97 | **rs35196866** | hsa-mir-4669 | 9q34.2 | 0.0 | 0.15 (Pilot CEU) | - | T:T | Genotyping failure |
| 98 | **seq_rs116932476** | hsa-mir-4479 | 9q34.3 | 98.9 | 0.033 (Pilot CEU) | 0.007 | G:A |  |
| 99 | **seq_rs62571442** | MIR3689 | 9q34.3 | 97.5 | 0.117 (Pilot CEU) | 0.404 | A:G |  |
| 100 | **rs2368392** | hsa-mir-604 | 10p11.23 | 99.4 | 0.246 | 0.289 | C:T |  |
| 101 | **rs11014002** | hsa-mir-603 | 10p12.2 | 0.0 | 0.064 | - | T:T | Genotyping failure |
| 102 | **rs11259096** | hsa-mir-1265 | 10p13 | 99.4 | 0.075 | 0.043 | T:C |  |
| 103 | **rs12780876** | hsa-mir-4293 | 10p13 | 98.6 | 0.288 | 0.306 | T:A |  |
| 104 | **rs7896283** | hsa-mir-4481 | 10p13 | 53.5 | 0.5 | 0.447 | A:G | Genotyping failure |
| 105 | **rs7070684** | hsa-mir-548aj-2 | 10p14 | 0.0 | 0.450 (Pilot CEU) | - | T:T | Genotyping failure |
| 106 | **rs2043556** | hsa-mir-605 | 10q21.1 | 98.6 | 0.186 | 0.205 | A:G |  |
| 107 | **rs4919510** | hsa-mir-608 | 10q24.31 | 99.7 | 0.177 | 0.178 | C:G |  |
| 108 | **rs7911488** | hsa-mir-1307 | 10q24.33 | 1.1 | 0.341 | - | A:G | Genotyping failure |
| 109 | **rs641071** | hsa-mir-4482 | 10q25.1 | 0.0 | 0.398 | - | T:T | Genotyping failure |
| 110 | **rs17091403** | hsa-mir-2110 | 10q25.3 | 100.0 | 0.097 | 0.089 | C:T |  |
| 111 | **rs12355840** | hsa-mir-202 | 10q26.3 | 83.6 | 0.167 (Pilot CEU) | 0.178 | T:C | No HWE Slovenia |
| 112 | **rs11032942** | hsa-mir-1343 | 11p13 | 0.0 | 0.125 | - | T:T | Genotyping failure |
| 113 | **rs75966923** | hsa-mir-4298 | 11p15.5 | 100.0 | 0.058 (Pilot CEU | 0.031 | C:A |  |
| 114 | **rs67042258** | hsa-mir-6128 | 11q12.1 | 98.9 | 0.242 (Pilot CEU) | 0.241 | G:A |  |
| 115 | **rs174561** | hsa-mir-1908 | 11q12.2 | 49.0 | 0.38 (Pilot CEU) | - | T:C | Genotyping failure |
| 116 | **rs12803915** | hsa-mir-612 | 11q13.1 | 98.6 | 0.267 | 0.171 | G:A |  |
| 117 | **rs515924** | hsa-mir-548al | 11q13.4 | 99.2 | 0.177 | 0.104 | A:G |  |
| 118 | **rs11237828** | hsa-mir-5579 | 11q14.1 | 0.0 | 0.225 | - | T:T | Genotyping failure |
| 119 | **rs35854553** | hsa-mir-3166 | 11q14.2 | 84.7 | 0.092 | 0.067 | A:T |  |
| 120 | **rs670637** | hsa-mir-3167 | 11q24.2 | 94.1 | 0.225 | 0.0 | T:T | Monomorphic |
| 121 | **seq_rs11048315** | MIR4302 | 12p12.1 | 99.4 | 0.175 (Pilot CEU) | 0.14 | G:A |  |
| 122 | **seq_rs117723462** | MIR3649 | 12p13.3 | 100.0 | 0.025 (Pilot CEU) | 0.001 | T:G |  |
| 123 | **rs215383** | hsa-mir-4494 | 12q13.11 | 99.2 | 0.125 | 0.157 | G:A |  |
| 124 | **rs832733** | hsa-mir-4698 | 12q13.11 | 0.0 | 0.333 | - | T:T | Genotyping failure |
| 125 | **rs11614913** | hsa-mir-196a-2 | 12q13.13 | 99.2 | 0.442 | 0.373 | C:T |  |
| 126 | **rs10878362** | hsa-mir-6074 | 12q14.3 | 0.0 | 0.358 | - | T:T | Genotyping failure |
| 127 | **rs2682818** | hsa-mir-618 | 12q21.31 | 98.3 | 0.208 (Pilot CEU) | 0.125 | C:A |  |
| 128 | **rs17022749** | hsa-mir-5700 | 12q22 | 0.0 | 0.068 | - | T:T | Genotyping failure |
| 129 | **rs2289030** | hsa-mir-492 | 12q22 | 99.7 | 0.08 | 0.088 | C:G |  |
| 130 | **rs17797090** | hsa-mir-3652 | 12q23.3 | 99.2 | 0.111 | 0.099 | G:A |  |
| 131 | **rs61938575** | hsa-mir-3922 | 12q23.3 | 82.4 | 0.367 | 0.278 | G:A |  |
| 132 | **rs7311975** | hsa-mir-1178 | 12q24.23 | 99.7 | 0.017 | 0.036 | T:C |  |
| 133 | **rs1055070** | hsa-mir-4700 | 12q24.31 | 99.7 | 0.033 (Pilot CEU) | 0.053 | T:G |  |
| 134 | **seq_rs111803974** | MIR3908 | 12q24.31 | 0.0 | 0.017 (Pilot CEU) | - | T:T | Genotyping failure |
| 135 | **rs1683709** | hsa-mir-3612 | 12q24.32 | 99.4 | 0.167 | 0.194 | C:T |  |
| 136 | **rs67976778** | hsa-mir-4305 | 13q14.11 | 0.0 | 0.325 (Pilot CEU) | - | T:T | Genotyping failure |
| 137 | **rs72631826** | hsa-mir-16-1 | 13q14.2 | 100.0 | 0.025 (miRNA panel) | 0.0 | T:T | Monomorphic |
| 138 | **rs1572687** | hsa-mir-5007 | 13q21.1 | 98.9 | 0.492 (Pilot CEU) | 0.464 | C:T |  |
| 139 | **rs2273626** | hsa-mir-4707 | 14q11.2 | 77.1 | 0.558 (Pilot CEU) | - | C:A | Genotyping failure |
| 140 | **seq_rs117650137** | hsa-mir-6717 | 14q11.2 | 99.7 | 0.017 (Pilot CEU) | 0.031 | G:A |  |
| 141 | **rs11156654** | mir-624 | 14q12 | 99.2 | 0.281 (Pilot CEU) | 0.246 | T:A |  |
| 142 | **rs35650931** | hsa-mir-6076 | 14q21.3 | 99.7 | 0.100 (Pilot CEU) | 0.105 | G:C |  |
| 143 | **rs28477407** | hsa-mir-4308 | 14q22.2 | 100.0 | 0.1 (Pilot CEU) | 0.084 | C:T |  |
| 144 | **rs12879262** | hsa-mir-4309 | 14q32.31 | 99.4 | 0.117 (Pilot CEU) | 0.155 | G:C |  |
| 145 | **rs12894467** | hsa-mir-300 | 14q32.31 | 99.7 | 0.392 (Pilot CEU) | 0.403 | C:T |  |
| 146 | **rs41286570** | hsa-mir-154 | 14q32.31 | 99.7 | 0.017 (Pilot CEU) | 0.0 | G:G | Monomorphic |
| 147 | **rs56103835** | hsa-mir-323b | 14q32.31 | 99.4 | 0.175 | 0.187 | T:C |  |
| 148 | **rs58834075** | hsa-mir-656 | 14q32.31 | 100.0 | 0.042 (Pilot CEU) | 0.025 | C:T |  |
| 149 | **rs61992671** | hsa-mir-412 | 14q32.31 | 98.3 | 0.467 (Pilot CEU) | 0.484 | G:A |  |
| 150 | **seq_rs111906529** | MIR299, MIR380 | 14q32.31 | 100.0 | 0.033 (Pilot CEU) | 0.016 | T:C |  |
| 151 | **rs3112399** | hsa-mir-4803 | 15q13.2 | 98.6 | 0.458 (Pilot CEU) | 0.461 | T:A | No HWE Spain |
| 152 | **seq_rs11269** | mir-1282 | 15q15.3 | 100.0 | 0.14 (CEPH) | 0.0 | G:G | Monomorphic |
| 153 | **rs2060455** | hsa-mir-4511 | 15q22.31 | 0.0 | 0.181 | - | T:T | Genotyping failure |
| 154 | **rs4414449** | hsa-mir-548ap | 15q25.3 | 68.0 | 0.325 | - | T:C | Genotyping failure |
| 155 | **rs4577031** | hsa-mir-548ap | 15q25.3 | 99.7 | 0.325 | 0.401 | A:T |  |
| 156 | **rs1439619** | hsa-mir-3175 | 15q26.1 | 85.0 | 0.45 (Pilot CEU) | 0.493 | A:C |  |
| 157 | **rs2910164** | hsa-mir-146a | 15q33.3 | 99.7 | 0.235 | 0.232 | G:C |  |
| 158 | **rs2291418** | hsa-mir-1229 | 15q35.3 | 100.0 | 0.05 | 0.03 | C:T |  |
| 159 | **rs897984** | hsa-mir-4519 | 16p11.2 | 0.0 | 0.283 (Pilot CEU) | - | T:T | Genotyping failure |
| 160 | **rs8054514** | hsa-mir-3176 | 16p13.3 | 99.4 | 0.105 | 0.151 | T:G |  |
| 161 | **rs7205289** | hsa-mir-140 | 16q22.1 | 92.6 | No freq/Bibliographic | 0.0 | C:C | Monomorphic |
| 162 | **rs7500280** | hsa-mir-4719 | 16q23.1 | 0.0 | 0.433 (Pilot CEU) | - | T:T | Genotyping failure |
| 163 | **rs74469188** | hsa-mir-6504 | 16q23.3 | 84.4 | 0.108 (Pilot CEU) | 0.116 | T:C |  |
| 164 | **rs35613341** | hsa-mir-5189 | 16q24.2 | 99.2 | 0.3 (Pilot CEU) | 0.329 | C:G | No HWE Spain |
| 165 | **rs56292801** | hsa-mir-5189 | 16q24.2 | 76.8 | 0.3 (Pilot CEU) | - | G:A | Genotyping failure |
| 166 | **rs12451747** | hsa-mir-1269b | 17p12 | 0.0 | 0.442 | - | T:T | Genotyping failure |
| 167 | **rs66507245** | hsa-mir-4731 | 17p12 | 0.0 | 0.45 (Pilot CEU) | - | T:T | Genotyping failure |
| 168 | **rs9913045** | hsa-mir-548h-3 | 17p12 | 0.0 | 0.342 (Pilot CEU) | 0.0 | T:T | Genotyping failure |
| 169 | **rs76800617** | hsa-mir-4521 | 17p13.1 | 99.2 | 0.333 (Pilot CEU) | 0.019 | A:G |  |
| 170 | **rs8078913** | hsa-mir-4520a | 17p13.1 | 90.4 | 0.548 | 0.453 | C:T |  |
| 171 | **rs2663345** | hsa-mir-3183 | 17p13.3 | 0.0 | 0.322 | - | T:T | Genotyping failure |
| 172 | **rs17885221** | hsa-mir-4733 | 17q11.2 | 99.4 | 0.025 (Pilot CEU) | 0.028 | C:T |  |
| 173 | **rs6505162** | hsa-mir-423 | 17q11.2 | 0.0 | 0.403 | - | T:T | Genotyping failure |
| 174 | **rs11651671** | hsa-mir-548at | 17q21.2 | 0.0 | 0.258 (Pilot CEU) | - | T:T | Genotyping failure |
| 175 | **rs7207008** | hsa-mir-2117 | 17q21.31 | 100.0 | 0.438 | 0.455 | T:A |  |
| 176 | **rs17759989** | hsa-mir-633 | 17q23.1 | 99.7 | 0.035 | 0.023 | A:G |  |
| 177 | **rs745666** | hsa-mir-3615 | 17q25.1 | 99.2 | 0.367 (Pilot CEU) | 0.386 | C:G |  |
| 178 | **rs73410309** | hsa-mir-4739 | 17q25.3 | 0.0 | 0.158 (Pilot CEU) | - | T:T | Genotyping failure |
| 179 | **rs72855836** | hsa-mir-3976 | 18p11.31 | 99.4 | 0.042 (Pilot CEU) | 0.054 | G:A | No HWE Slovenia |
| 180 | **rs7227168** | hsa-mir-4741 | 18q11.2 | 99.4 | 0.111 (Pilot CEU) | 0.127 | C:T |  |
| 181 | **rs41274312** | hsa-mir-187 | 18q12.2 | 99.4 | 0.017 (Pilot CEU) | 0.007 | G:A |  |
| 182 | **rs78396863** | hsa-mir-4743 | 18q21.1 | 99.4 | 0.025 (Pilot CEU) | 0.016 | G:C |  |
| 183 | **rs12456845** | hsa-mir-4744 | 18q21.2 | 99.7 | 0.04 | 0.037 | T:C |  |
| 184 | **rs41292412** | hsa-mir-122 | 18q21.31 | 99.4 | 0.025 (Pilot CEU) | 0.001 | C:T |  |
| 185 | **rs7247237** | hsa-mir-3188 | 19p13.11 | 98.6 | 0.225 | 0.333 | C:T |  |
| 186 | **rs895819** | mir-27a | 19p13.12 | 1.4 | No freq/Bibliographic | - | T:C | Genotyping failure |
| 187 | **rs2967897** | hsa-mir-5695 | 19p13.13 | 100.0 | 0.333 (Pilot CEU) | 0.0 | G:G | Monomorphic |
| 188 | **rs72996752** | hsa-mir-4999 | 19p13.2 | 89.5 | 0.175 (Pilot CEU) | 0.242 | A:G |  |
| 189 | **rs10422347** | hsa-mir-4745 | 19p13.3 | 98.9 | 0.092 (Pilot CEU) | 0.09 | C:T |  |
| 190 | **rs10406069** | hsa-mir-5196 | 19q13.12 | 98.6 | 0.212 | 0.187 | G:A |  |
| 191 | **rs8667** | hsa-mir-4751 | 19q13.33 | 95.8 | 0.37 | 0.401 | G:A |  |
| 192 | **rs4112253** | hsa-mir-4751 | 19q13.42 | 98.6 | 0.35 | 0.339 | C:G |  |
| 193 | **rs57111412** | hsa-mir-1283-1 | 19q13.42 | 0.0 | 0.067 (Pilot CEU) | - | T:T | Genotyping failure |
| 194 | **rs71363366** | hsa-mir-1283-2 | 19q13.42 | 98.9 | 0.017 (Pilot CEU) | 0.037 | C:G |  |
| 195 | **rs74704964** | hsa-mir-518d | 19q13.42 | 83.6 | 0.067 (Pilot CEU) | 0.029 | C:T |  |
| 196 | **rs75598818** | hsa-mir-520f | 19q13.42 | 99.4 | 0.042 (Pilot CEU) | 0.027 | G:A |  |
| 197 | **seq_rs112328520** | MIR520G | 19q13.42 | 99.2 | 0.017 (Pilot CEU) | 0.07 | C:T |  |
| 198 | **rs72502717** | hsa-mir-3689f | 19q34.3 | 0.0 | 0.117 (Pilot CEU) | - | T:T | Genotyping failure |
| 199 | **rs11907020** | hsa-mir-3192 | 20p11.23 | 99.4 | 0.028 | 0.019 | T:C |  |
| 200 | **rs3746444** | hsa-mir-499a | 20q11.22 | 99.7 | 0.175 (Pilot CEU) | 0.179 | T:C |  |
| 201 | **seq_rs113808830** | MIR4532 | 20q13.2 | 98.6 | 0.025 (Pilot CEU) | 0.108 | C:T |  |
| 202 | **rs73177830** | hsa-mir-4532 | 20q13.32 | 0.0 | 0.033 (Pilot CEU) | - | T:T | Genotyping failure |
| 203 | **seq_rs117258475** | MIR296 | 20q13.32 | 99.2 | 0.025 (Pilot CEU) | 0.01 | G:A |  |
| 204 | **rs4809383** | hsa-mir-941-1 | 20q13.33 | 84.1 | 0.042 (Pilot CEU) | 0.133 | C:T |  |
| 205 | **rs6062431** | hsa-mir-4326 | 20q13.33 | 98.9 | 0.392 (Pilot CEU) | 0.367 | G:C | No HWE Spain |
| 206 | **rs6513496** | hsa-mir-646 | 20q13.33 | 99.4 | 0.175 (Pilot CEU) | 0.199 | T:C |  |
| 207 | **rs73147065** | hsa-mir-647 | 20q13.33 | 0.0 | 0.117 (Pilot CEU) | - | T:T | Genotyping failure |
| 208 | **rs4822739** | hsa-mir-548j | 22q12.1 | 100.0 | 0.045 | 0.052 | C:G |  |
| 209 | **rs5997893** | hsa-mir-3928 | 22q12.2 | 99.7 | 0.354 | 0.344 | G:A |  |
| 210 | **rs60308683** | hsa-mir-4762 | 22q13.31 | 0.0 | 0.1 | - | T:T | Genotyping failure |
| 211 | **rs72631825** | hsa-mir-222 | Xp11.3 | 0.0 | 0.019 (miRNA panel) | - |  | Genotyping failure |
| 212 | **rs5965660** | hsa-mir-888 | Xq27.3 | 0.0 | 0.161 | - |  | Genotyping failure |
| 213 | **rs72631816** | hsa-mir-105-2 | Xq28 | 0.0 | 0.011 (miRNA panel) | - |  | Genotyping failure |

| **Supplementary Table S2.** Genotype association studies in the Spanish and Slovenian populations. | | | | | | |  |
| --- | --- | --- | --- | --- | --- | --- | --- |
|  | **SNP** | **miRNA** | **Genotype** | **Controls** | **Cases** | **OR (95%CI)** | **P_Global_** |
|  |  |  |  | **(N=256)** | **(N=94)** |  |  |
| 1 | rs77639117 | mir-576 | AA | 249 (97.3) | 81 (88.0) |  | 0.001 |
|  | (pre-miRNA) |  | AT | 7 (2.7) | 10 (10.9) | AT/TT 4.83 (1.81-12.87)(dom) |  |
|  |  |  | TT | 0 (0.0) | 1 (1.1) |  |  |
| 2 | rs9877402 | mir-5682 | AA | 233 (92.5) | 32 (84.2) |  | 0.002 |
|  | (pre-miRNA) |  | AG | 19 (7.5) | 3 (7.9) | GG 0 (rec) |  |
|  |  |  | GG | 0 (0.0) | 3 (7.9) |  |  |
| 3 | rs2273626 | mir-4707 | AA | 53 (29.9) | 13 (14.4) |  | 0.004 |
|  | (seed) |  | AC | 79 (44.6) | 47 (52.2) | AA 0.40 (0.20-0.77) (rec) |  |
|  |  |  | CC | 45 (25.4) | 30 (33.3) |  |  |
| 4 | rs4674470 | mir-4268 | TT | 151 (59.2) | 67 (75.3) |  | 0.006 |
|  | (pre-miRNA) |  | CT | 87 (34.1) | 19 (21.3) | CT/CC 0.48 (0.28-0.82)(dom) |  |
|  |  |  | CC | 17 (6.7) | 3 (3.4) |  |  |
| 5 | rs12894467 | mir-300 | CC | 99 (38.7) | 26 (28.0) |  | 0.01 |
|  | (pre-miRNA) |  | CT | 124 (48.4) | 45 (48.4) | TT 1.57 (1.11-2.22) (log) |  |
|  |  |  | TT | 33 (12.9) | 22 (23. 7) |  |  |
| 6 | rs10422347 | mir-4745 | CC | 218 (85.8) | 68 (74.7) |  | 0.02 |
|  | (miRNA) |  | CT | 34 (13.4) | 23 (25.3) | CT/TT 2.05 (1.14-3.69)(dom) |  |
|  |  |  | TT | 2 (0.8) | 0 (0.0) |  |  |
| 7 | rs2070960 | mir-3620 | CC | 216 (85.0) | 74 (82.2) |  | 0.02 |
|  | (seed) |  | CT | 38 (15.0) | 13 (14.4) | TT 0 (rec) |  |
|  |  |  | TT | 0 (0.0) | 3 (3.3) |  |  |
| 8 | rs7247237 | mir-3188 | CC | 115 (45.3) | 37 (40.2) |  | 0.03 |
|  | (pre-miRNA) |  | CT | 121 (47.6) | 41 (44.6) | TT 2.35 (1.12-4.95)(rec) |  |
|  |  |  | TT | 18 (7.1) | 14 (15.2) |  |  |
| 9 | rs2910164 | mir-146a | GG | 144 (56.2) | 64 (68.8) |  | 0.03 |
|  | (pre-miRNA) |  | CG | 96 (37.5) | 23 (24.7) | CG/CC 0.58 (0.35-0.96)(dom) |  |
|  |  |  | CC | 16 (6.2) | 6 (6.5) |  |  |
| 10 | rs4112253 | mir-4752 | CC | 103 (40.2) | 46 (52.3) |  | 0.03 |
|  | (pre-miRNA) |  | CG | 119 (46.5) | 35 (39.8) | GG 0.67 (0.46-0.98)(rec) |  |
|  |  |  | GG | 34 (13.3) | 7 (8.0) |  |  |
| 11 | rs67042258 | mir-6128 | GG | 143 (56.1) | 59 (65.6) |  | 0.04 |
|  | (pre-miRNA) |  | AG | 97 (38.0) | 22 (24.4) | AG 0.55 (0.32-0.96) |  |
|  |  |  | AA | 15 (5.9) | 9 (10.0) | AA 1.45 (0.60-3.51)(codom) |  |

**Abbreviations: OR Odd Ratio, CI Confidence Interval**

**Supplementary Table S3**: The enriched pathways by mir-412 overrepresented in KEGG and Biocarta enrichment analysis pathway tools.

| Pathway name | Set size | Candidates contained | p-value | q-value | Pathway source |
| --- | --- | --- | --- | --- | --- |
| Wnt signaling pathway | [143](http://cpdb.molgen.mpg.de/CPDB/showSetDetails?sp=p&st=0) | [8 (5.6%)](http://cpdb.molgen.mpg.de/CPDB/showSetDetails?sp=p&st=0) | 0.000829 | 0.105 | **KEGG** |
| wnt signaling pathway | [32](http://cpdb.molgen.mpg.de/CPDB/showSetDetails?sp=p&st=1) | [4 (12.5%)](http://cpdb.molgen.mpg.de/CPDB/showSetDetails?sp=p&st=1) | 0.000938 | 0.105 | **BioCarta** |

**Supplementary Table S4:** Genes of Ca2+ signaling pathway targeted by mir-656.

| *Entrez-gene ID* | *entrez-gene name* |
| --- | --- |
| [51701](http://www.ncbi.nlm.nih.gov/gene?term=51701) | *NLK* : nemo like kinase |
| [54331](http://www.ncbi.nlm.nih.gov/gene?term=54331) | *GNG2* : G protein subunit gamma 2 |
| [5592](http://www.ncbi.nlm.nih.gov/gene?term=5592) | *PRKG1* : protein kinase, cGMP-dependent, type I |
| [8323](http://www.ncbi.nlm.nih.gov/gene?term=8323) | *FZD6* : frizzled class receptor 6 |
| [7976](http://www.ncbi.nlm.nih.gov/gene?term=7976) | *FZD3* : frizzled class receptor 3 |
| [23236](http://www.ncbi.nlm.nih.gov/gene?term=23236) | *PLCB1* : phospholipase C beta 1 |
| [7855](http://www.ncbi.nlm.nih.gov/gene?term=7855) | *FZD5* : frizzled class receptor 5 |

**Supplementary Table S5**: The enriched pathways by mir-412 and mir-656.

| **Pathway name** | Set size | Candidates contained | p-value | q-value | Pathway source |
| --- | --- | --- | --- | --- | --- |
| **Wnt signaling pathway** | [143](http://cpdb.molgen.mpg.de/CPDB/showSetDetails?sp=p&st=0) | [16 (11.2%)](http://cpdb.molgen.mpg.de/CPDB/showSetDetails?sp=p&st=0) | 0.000177 | 0.0714 | **KEGG** |
| **wnt signaling pathway** | [32](http://cpdb.molgen.mpg.de/CPDB/showSetDetails?sp=p&st=1) | [7 (21.9%)](http://cpdb.molgen.mpg.de/CPDB/showSetDetails?sp=p&st=1) | 0.000213 | 0.0714 | **BioCarta** |
| **Pathways in cancer** | [397](http://cpdb.molgen.mpg.de/CPDB/showSetDetails?sp=p&st=2) | [31 (7.8%)](http://cpdb.molgen.mpg.de/CPDB/showSetDetails?sp=p&st=2) | 0.000262 | 0.0714 | **KEGG** |
| **Retrograde endocannabinoid signaling** | [101](http://cpdb.molgen.mpg.de/CPDB/showSetDetails?sp=p&st=3) | [12 (11.9%)](http://cpdb.molgen.mpg.de/CPDB/showSetDetails?sp=p&st=3) | 0.00065 | 0.133 | **KEGG** |
| **Amino acid and oligopeptide SLC transporters** | [51](http://cpdb.molgen.mpg.de/CPDB/showSetDetails?sp=p&st=4) | [8 (15.7%)](http://cpdb.molgen.mpg.de/CPDB/showSetDetails?sp=p&st=4) | 0.000831 | 0.136 | **Reactome** |

**Supplementary Table S6:** Genes of WNT signaling pathway targeted by mir-412 and mir-656

| *Entrez-gene ID* | *entrez-gene name* |
| --- | --- |
| [**595**](http://www.ncbi.nlm.nih.gov/gene?term=595) | *CCND1* : cyclin D1 |
| [**8323**](http://www.ncbi.nlm.nih.gov/gene?term=8323) | *FZD6* : frizzled class receptor 6 |
| [**7855**](http://www.ncbi.nlm.nih.gov/gene?term=7855) | *FZD5* : frizzled class receptor 5 |
| [**23236**](http://www.ncbi.nlm.nih.gov/gene?term=23236) | *PLCB1* : phospholipase C beta 1 |
| [**8945**](http://www.ncbi.nlm.nih.gov/gene?term=8945) | *BTRC* : beta-transducin repeat containing E3 ubiquitin protein ligase |
| [**6422**](http://www.ncbi.nlm.nih.gov/gene?term=6422) | *SFRP1* : secreted frizzled related protein 1 |
| **[10023](http://www.ncbi.nlm.nih.gov/gene?term=10023)** | *FRAT1* : FRAT1, WNT signaling pathway regulator |
| **[7976](http://www.ncbi.nlm.nih.gov/gene?term=7976)** | *FZD3* : frizzled class receptor 3 |
| [**7473**](http://www.ncbi.nlm.nih.gov/gene?term=7473) | *WNT3* : Wnt family member 3 |
| [**79718**](http://www.ncbi.nlm.nih.gov/gene?term=79718) | *TBL1XR1* : transducin beta like 1 X-linked receptor 1 |
| **[894](http://www.ncbi.nlm.nih.gov/gene?term=894)** | *CCND2* : cyclin D2 |
| [**81839**](http://www.ncbi.nlm.nih.gov/gene?term=81839) | *VANGL1* : VANGL planar cell polarity protein 1 |
| [**11197**](http://www.ncbi.nlm.nih.gov/gene?term=11197) | *WIF1* : WNT inhibitory factor 1 |
| [**4040**](http://www.ncbi.nlm.nih.gov/gene?term=4040) | *LRP6* : LDL receptor related protein 6 |
| **[51701](http://www.ncbi.nlm.nih.gov/gene?term=51701)** | *NLK* : nemo like kinase |
| [**4089**](http://www.ncbi.nlm.nih.gov/gene?term=4089) | *SMAD4* : SMAD family member 4 |

**Supplementary Figure S1.** Secondary structures of miRNAs showing significant SNPs in the Spanish population.


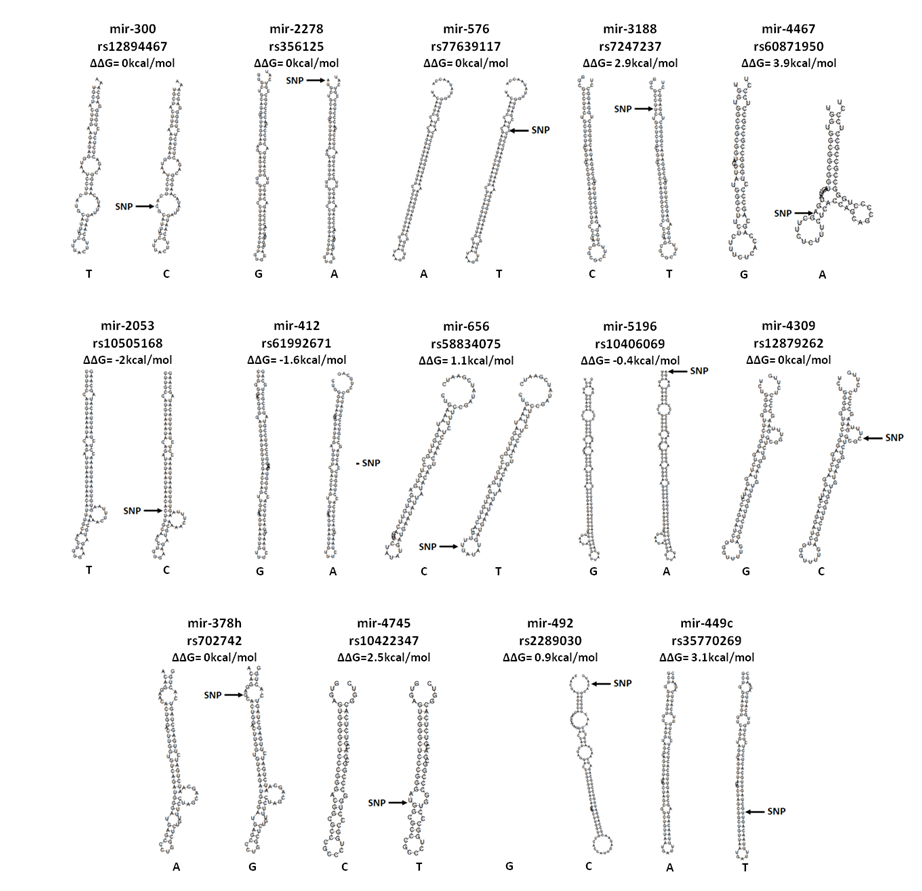


**Supplementary Figure S2.** Secondary structures of miRNAs showing significant SNPs in the Slovenian population.


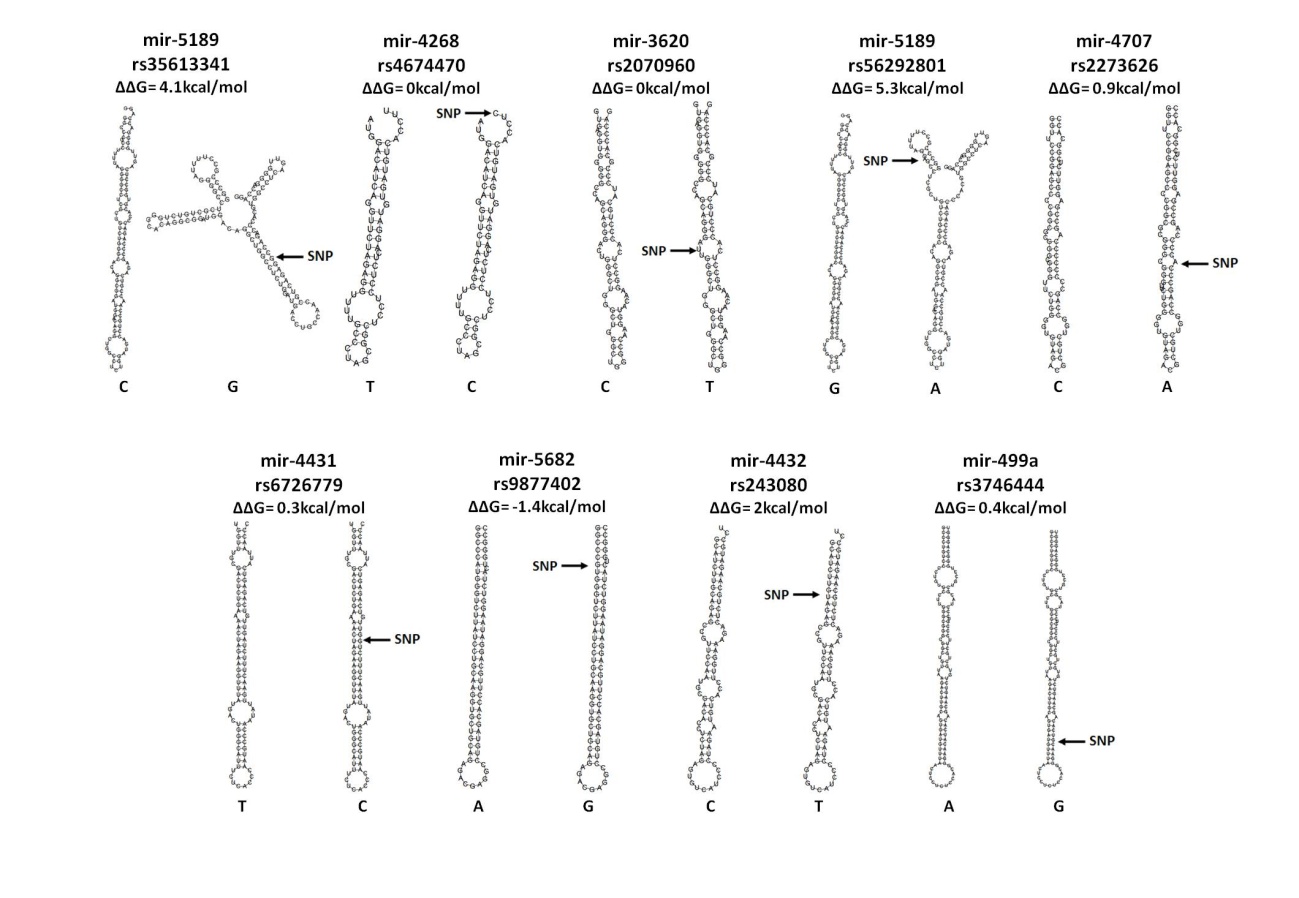


**Supplementary Figure S3.** Secondary structures of the three new miRNAs showing significant SNPs in the Spanish and Slovenian populations together.


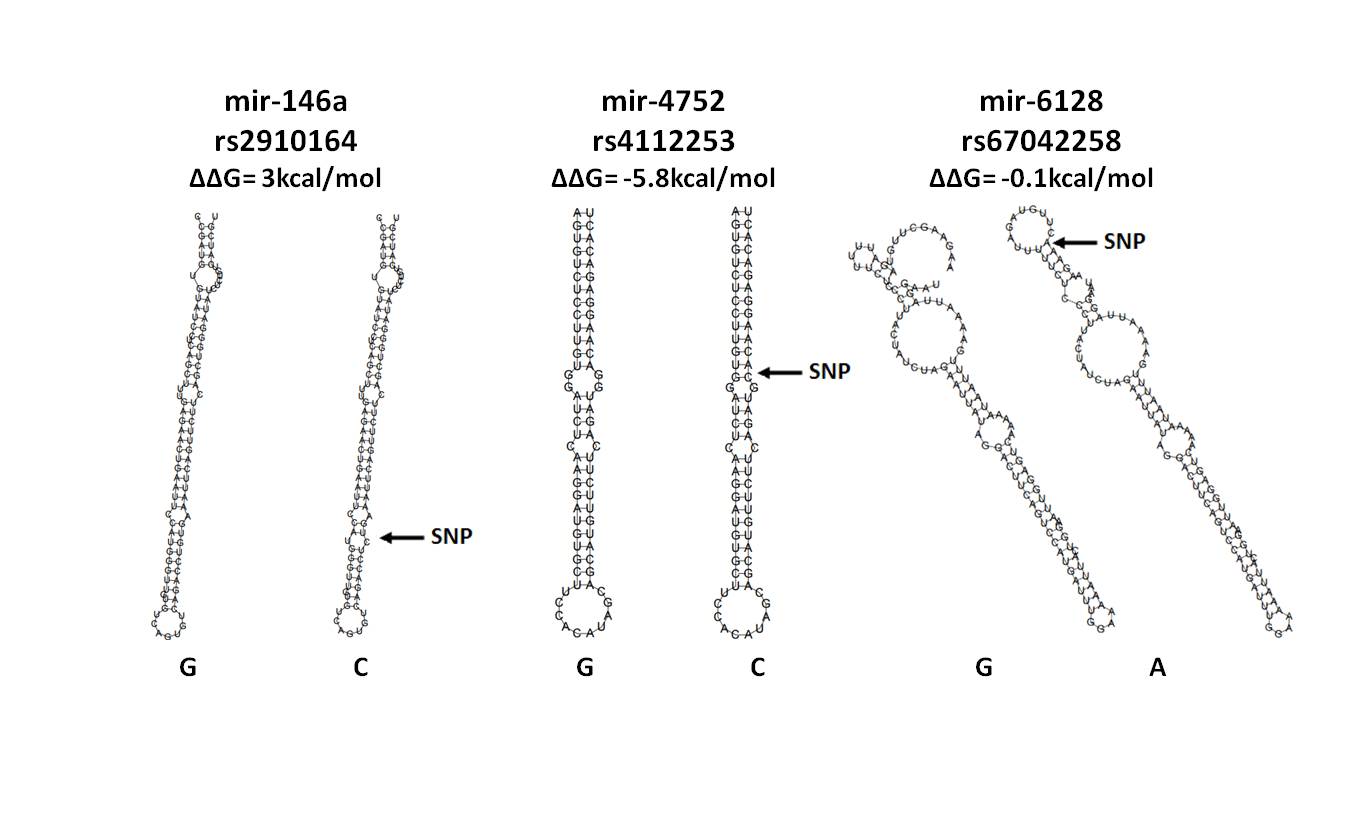


**Supplementary Figure S4.** Forest plot for rs2910164.


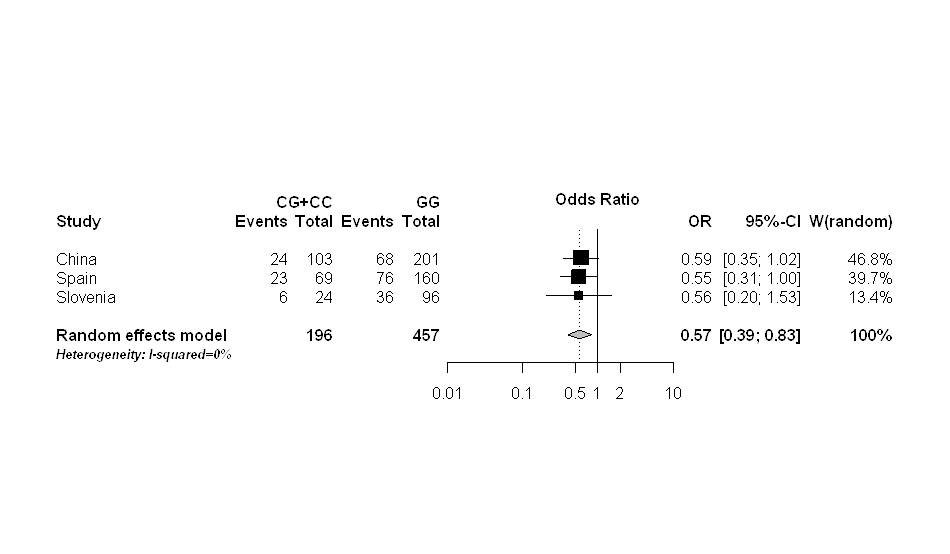

Supplement: Supplementary file 1 — Supplementary information [file 41598_2018_33712_MOESM1_ESM.docx]
